# An observational study to determine the effect of delayed admission to the intensive care unit on patient outcome

**DOI:** 10.1186/cc11650

**Published:** 2012-10-01

**Authors:** David JP O'Callaghan, Parveen Jayia, Eyston Vaughan-Huxley, Michael Gribbon, Maie Templeton, James RA Skipworth, Anthony C Gordon

**Affiliations:** 1 Section of Anaesthetics, Pain Medicine and Intensive Care, Imperial College London, Chelsea and Westminster Hospital Campus, 369 Fulham Road, London SW10 9NH, UK; 2 Department of Critical Care, 11th Floor, Imperial College Healthcare NHS Trust, Charing Cross Hospital, Fulham Palace Road, London W6 8RF, UK; 3 Department of Surgical and Interventional Science, Medical School Building, 74 Huntley Street, UCL, London, WC1E 6DH, UK

## Abstract

**Introduction:**

Delayed patient admission to the intensive care unit (ICU) due to lack of bed availability is a common problem, but the effect on patient outcome is not fully known.

**Methods:**

A retrospective study was performed using departmental computerised records to determine the effect of delayed ICU admission and temporary management within the operating theatre suite on patient outcome. Emergency surgical and medical patients admitted to the ICU (2003 to 2007) were divided into delay (more than three hours from referral to admission) and no-delay (three or fewer hours from referral to admission) groups. Our primary outcome measure was length of ICU stay. Secondary outcome measures were mortality rates and duration of organ support.

**Results:**

A total of 1,609 eligible patients were included and 149 (9.3%) had a delayed admission. The delay and no-delay groups had similar baseline characteristics. Median ICU stay was 5.1 days (delay) and 4.5 days (no-delay) (*P *= 0.55) and ICU mortality was 26.8% (delay) and 24.2% (no-delay) (*P *= 0.47). Following adjustment for demographic and baseline characteristics there was no difference in either length of ICU stay or mortality rates between groups. ICU admission delay was associated with both an increased requirement for advanced respiratory support (92.3% delay vs. 76.4% no-delay, *P *<0.01) and a longer time spent ventilated (median four days delay vs. three days no-delay, *P *= 0.04).

**Conclusions:**

No significant difference in length of ICU stay or mortality rate was demonstrated between the delay and no-delay cohorts. Patients within the delay group had a significantly greater requirement for advanced respiratory support and spent a longer time ventilated.

## Introduction

Demand for critical care services is increasing worldwide [1] and there are large variations in Intensive Care Unit (ICU) bed provision between countries. In the United Kingdom (UK) this figure remains low and compares unfavourably with other nations [2] despite recent increases in absolute bed numbers [3]. This was highlighted by the problems faced during the recent winter flu surge in which operating theatres and recovery areas were adapted in order to cope with the extra demand for ICU beds [4].

The incidence of severe sepsis, the most common cause for general ICU admission, is increasing [5,6] with forecasts suggesting that this will continue, due to an ageing population [7] and that this will increase pressure on pre-existing ICU beds. Bed availability is further hindered by the increasing number of ICU discharges that are delayed due to a shortage of general ward beds [3]. These factors contribute to a situation whereby an ICU bed may not be immediately available when a patient requires admission. In one UK study, 14% of ICU referrals were refused due to a lack of ICU beds [8] with 65% of UK intensive care specialists reporting ICU admissions to be limited by bed availability [9].

The net result of these processes is that difficult decisions about patient admission and transfer will become increasingly common. The process of transfer for non-clinical reasons (that is, for comparable care rather than for specialist care) can often leave the clinician facing a dilemma [10]: should they transfer the new potentially unstable patient to an alternative centre or relocate a more physiologically stable existing patient? Transfer itself can be associated with adverse events and deterioration in patient physiology [11,12], with inter-hospital transfer associated with a worse outcome than remaining within an institution [13]. In the UK much of the data collection surrounding transfers is inadequate [14] so that accurately quantifying risk/benefit ratio is difficult.

The alternative to inter-hospital transfer is to manage the new patient on site until an ICU bed becomes available. This is the policy employed within Charing Cross Hospital where the operating theatre suite is often used to provide a suitably monitored environment in which to manage the patient. However, this policy of "boarding" a patient rather than transferring out can result in a delayed ICU admission. There is currently no UK-based study and a general paucity of evidence examining the impact of both this specific holding strategy or of a delayed ICU admission on patient outcome.

We, therefore, examined the effect of a delayed admission to ICU and of this specific management strategy of "boarding" patients in the operating theatre suite on outcomes as determined by length of ICU stay, mortality rates and duration of organ support.

## Materials and methods

### Study

Ethics committee approval was waived for this study as it involved retrospective analysis of anonymous, routinely collected, group data.

Departmental computerised patient records were analysed to identify eligible patients admitted to the ICU between 1 January 2003 and 31 December 2007. During this period, Charing Cross Hospital ran between 475 and 582 beds with a 12-bed ICU admitting adult medical and surgical patients, including neurosciences. There were four full-time intensive care specialists in 2003, this increased to five in 2004 but remained constant for the remainder of the study period. The hospital has an Emergency Department but no paediatric, cardiothoracic surgery, or obstetrics and gynaecology services. The hospital underwent a merger at the end of 2007 and the ICU expanded at this time; hence, this was used as the study endpoint.

A delayed admission was defined according to our regional critical care network guidelines as taking greater than three hours from the point of acceptance by the critical care team to patient arrival on the ICU. Time spent for patient investigation and/or treatment was not classified as delay. Readmissions, elective surgical admissions and inter-hospital transfers into the ICU were excluded. Patients readmitted to the ICU during the same hospital admission were classed as readmissions and those receiving a planned surgical procedure (even if the ICU admission was not planned) were classed as elective surgical admissions.

### Data collection and analysis

Demographic data, including age, sex, diagnosis, admission category (medical or surgical), Acute Physiology and Chronic Health Evaluation (APACHE) II score [15] and source of admission, were collected. The primary reason for ICU admission, as well as physiological and laboratory data for the first 24 hours after ICU referral, were also recorded.

Recorded outcome variables for all patients were length of ICU stay, and ICU and hospital mortality. The UK critical care minimum dataset [16] was introduced on 1 April 2006 and daily organ support data were collected from this date onwards. The definitions of organ support are listed in Table 1.

**Table 1:** Organ support categories as defined by critical care minimum data set, January 2006

*Basic respiratory support*	More than 50% oxygen delivered by face mask Close observation due to potential for acute deterioration Physiotherapy or suction to clear secretions at least two hourly Patients recently extubated after a prolonged period of intubation and mechanical ventilation Mask CPAP or non-invasive ventilation ETT but no support
*Advanced respiratory support*	Invasive mechanical ventilatory support BiPAP or CPAP applied via a tracheal tube Extracorporeal respiratory support

*Basic cardiovascular support*	Treatment of circulatory instability due to hypovolaemia Use of a CVP line Use of an arterial line Single intravenous vasoactive drug Intravenous drugs to control cardiac arrhythmias Non-invasive measurement of cardiac output

*Advanced cardiovascular support*	Multiple intravenous vasoactive and/or rhythm controlling drugs Patients resuscitated after cardiac arrest where intensive therapy is considered clinically appropriate Observation of cardiac output and derived indices Intra aortic balloon pumping Insertion of a temporary cardiac pacemaker Placement of a gastrointestinal tonometer

*Renal support*	Acute renal replacement therapy

CPAP, continuous positive airway pressure; CVP, central venous pressure; ETT. endo-tracheal tube; BiPAP, bi-level positive airway pressure

Patient data were extracted from the hospital database and analysed using Microsoft Excel 2010 (Microsoft, Redmond, Washington USA) and Statistical Package for the Social Sciences version 19 (IBM, Armonk, New York, USA). Continuous variables were analysed using the Student's *t-*test or Mann-Whitney U test as appropriate. Categorical data were analysed using a Chi-squared test. Multivariate regression models were used to adjust for baseline characteristics (including age, sex, APACHE II score, delay admission category and other variables that were significantly different between the two groups) when examining the effect of delayed admission on length of ICU stay and ICU mortality. A *P*-value of less than 0.05 was considered statistically significant.

## Results

**Figure 1:**
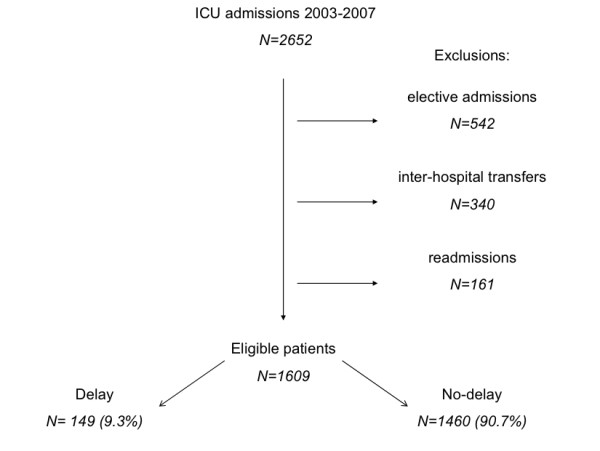
**Patient flow**.

Between January 2003 and December 2007, 2,652 patients were admitted to Charing Cross Hospital ICU. Of these, 1,609 met the inclusion criteria (Figure 1) and 149 patients (9.3%) had a delayed admission. The annual rates of delayed admission were 4.9% (13/268) in 2003, 10.9% (33/302) in 2004, 14.1% (48/341) in 2005, 5.2% (18/349) in 2006 and 10.1% (37/349) in 2007. For all patients, ICU survival status and length of ICU stay was known but hospital survival status was not recorded for 16 patients. There were 598 patients admitted to ICU from 1 April 2006 to 31 December 2007 who also had daily organ support data collected.

Demographic data were similar between the delayed and non-delayed groups (Table 2), other than a trend towards lower age within the delay group (55 ± 17 years) compared to the no-delay group (57 ± 19 years, *P *= 0.07). The median duration of delay was 6 hours (IQR 4.5 to 10 hours). Within the delay group 28 patients (18.8%) were admitted directly from the ward, 11 (7.4%) from the Emergency Department (ED) and 110 (73.8%) from the theatre suite. Only 32 patients (21.5%) in the delay group had undergone an operative intervention, which was significantly less than the no-delay group (30.5%, *P *= 0.02). The other 78 patients admitted to ICU from the theatre suite were transferred there from elsewhere in the hospital for continued management pending ICU admission. The delay group had a significantly higher percentage of patients with respiratory failure as a cause for admission (25.5% vs. 15.6%, *P *<0.01) than the no-delay group and a lower partial pressure of oxygen in the blood (PaO_2_):fraction of inspired oxygen (FiO_2_) ratio (25.8 ± 15.1 vs. 29.6 ± 18.0, *P *= 0.02) and a lower Glasgow Coma Scale (GCS) (6, IQR 3 to 15 vs. 10, IQR 3 to 15, *P *= 0.03) in the first 24 hours after ICU referral.

**Table 2:** Demographic and baseline characteristics for delay and no-delay groups

	Delay*N = 149*	No-Delay*N = 1,460*	*P *- value
Age	55 ± 17	57 ± 19	0.07

Male sex	94 (63.1)	902 (61.8)	0.64

**Referring specialty:**			
Medical	86 (57.3)	833 (57.1)	0.88
Surgical	63 (42.3)	627 (42.9)	

APACHE II score	20 (15 to 26)	20 (1 to 26)	0.99

***Comorbidities:***			
COPD/Asthma	17 (11.4)	142 (9.7)	0.51
Ischaemic heart disease	15 (10.1)	148 (10.1)	0.98
End stage renal failure	5 (3.4)	35 (2.4)	0.48
Metastatic cancer	7 (4.7)	47 (3.2)	0.34
Acquired immunosuppression	7 (4.7)	82 (5.6)	0.64
***Cause for ICU admission:***			
Cardiac arrest/failure	18 (12.1)	117 (8)	0.08
Haemorrhage	9 (6)	133 (9.1)	0.21
Operative intervention	32 (21.5)	446 (30.5)	0.02
Respiratory failure	38 (25.5)	230 (15.6)	<0.01
Sepsis	23 (15.4)	202 (13.8)	0.6
Trauma	7 (4.7)	70 (4.8)	0.96
Neurological failure	5 (3.4)	108 (7.4)	0.07
Other	17 (11.4)	154 (10.5)	0.75

***Recorded data in the first 24 hours after ICU referral:***			
Intubated	98 (66)	847 (58)	0.07
Lowest PaO_2_:FiO_2 _(kPa)	25.8 (± 15.1)	29.6 (± 18)	0.02
Mean arterial pressure (mmHg)	80.2 (± 33.1)	79.1 (± 32.3)	0.71
Creatinine (μmol/l)	91 (73.2 to 143)	94 (72 to 147)	0.69
Lowest bicarbonate (mmol/l)	19.4 (± 4.4)	19.6 (± 8.1)	0.72
GCS	6 (3 to 15)	10 (3 to 15)	0.03
Bilirubin (μmol/l)	22.7 (± 31.9)	20.3 (± 31)	0.41
Haemoglobin (g/l)	11.2 (± 2.1)	11.4 (± 1.9)	0.2

Data shown as N (%), mean (± SD) or median (IQR)

**Figure 2:**
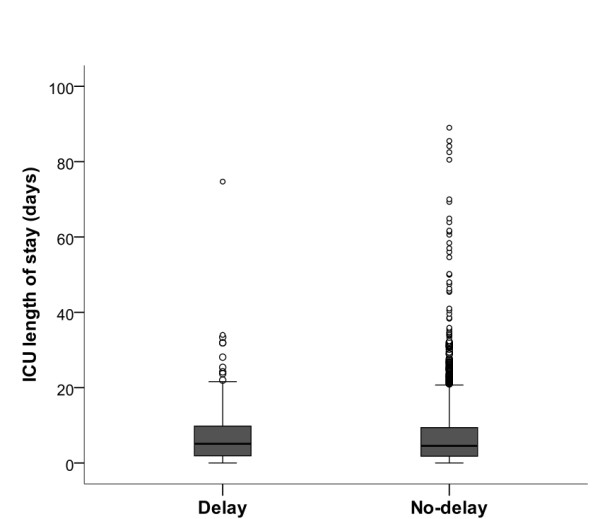
**Length of ICU stay**. Components: Black line = median; Box margins = 25^th ^and 75^th ^percentile; Whiskers = x1.5 of inter-quartile range (IQR); Circles = outliers (> × 1.5 of IQR).

There was no significant difference in length of ICU stay between the delay group (median 5.1 days, IQR 1.9 to 9.8) and no-delay group (median 4.5 days, IQR = 1.8 to 9.4, *P *= 0.55) (Figure 2). There was no significant difference in ICU mortality (delay = 26.8% (40/149) vs. no-delay = 24.2% (353/1,460); *P *= 0.47) or hospital mortality (delay = 36.2% (54/149); no-delay = 32.8% (479/1,460); *P *= 0.44) between the groups (Table 2).

We performed multivariate regression analysis to adjust for any imbalances at baseline between the delay and no-delay groups. Using linear regression analysis, delayed admission was not associated with length of ICU stay (β 0.48, 95% CI -1.5 to 2.46, *P *= 0.64, Table 3). Similarly, on binary logistic regression analysis, there was no association between ICU mortality (OR 1.27, 95% CI 0.81 to 2.0, *P *= 0.29,Table 4) and delayed admission. Increasing age, male sex and operative intervention were associated with increased ICU stay (Table 3). Increasing APACHE II score was associated with significantly higher ICU mortality (Table 4).

**Table 3:** Linear regression analysis

*Covariates*	*β (slope)*	*95% CI of β*	*P-value*
Age	0.04	0.03 to 0.07	0.03

APACHE II	0.06	-0.02 to 0.13	0.12

Male sex	1.28	0.09 to 2.46	0.04

Respiratory failure	1.48	-1.42 to 3.1	0.07

Operative intervention	1.5	0.13 to 2.87	0.03

Delayed admission	0.48	-1.5 to 2.46	0.64

Linear regression analysis: dependent variable is length of ICU stay in days.

For continuous variables (age and APACHE II score) β refers to incremental increase in age/score.

**Table 4:** Binary regression analysis

*Covariates*	*Odds ratio*	*95% CI *	*P-value*
Age	0.995	0.99 to 1.0	0.22

APACHE II	1.15	1.13 to 1.17	<0.001

Male sex	0.91	0.68 to 1.21	0.5

Respiratory failure	1.03	0.65 to 1.46	0.9

Operative intervention	1.24	0.87 to 1.75	0.23

Delayed admission	1.27	0.81 to 2.00	0.29

Binary logistic regression analysis: dependent variable is ICU mortality.

For continuous variables (age and APACHE II score) OR refers to incremental increase in age/score.

There was no association between length of delay in hours and mortality. Within the delay group, the odds ratio for ICU mortality was 0.97 for each hour delay (95% CI 0.89 to 1.05, *P *= 0.41). We further tested if the longest delays affected outcome (those patients in the highest quartile of delay, >10 hours). There was no difference in the length of ICU stay in the longest delayed admissions compared to no-delay patients (median 5.2 vs. 4.6 days respectively, *P *= 0.61) or compared to those whose delay was in the lowest (<4.5 hours) quartile (median 5.2 days - highest quartile vs. 5.1 days - lowest quartile, *P *= 0.79). Similarly, the ICU mortality rate was not increased at 20.1% (7/34), in the longest delayed patients.

The majority of patients (73.8%) in the delayed admission group were managed in the operating theatre suite pending ICU admission. There was no difference in length of ICU stay between those managed in the theatre suite compared to those managed in the ED/wards during the delay period (median 4.8 vs. 5.8 days respectively, *P *= 0.36). However, there was a trend toward a higher ICU mortality in patients not managed within the theatre suite during the delay (ED/wards 38.5% mortality (15/39) vs. theatre suite 22.7% mortality (25/110), *P *= 0.06) that persisted after adjusting for baseline characteristics (OR 2.94, 95% CI 0.89 to 6.46, *P *= 0.08).

There was a trend toward patients in the delay group having higher rates of intubation during the first 24 hours after ICU referral (66% delayed vs. 58% non-delayed, *P *= 0.07, Table 2) and the delay group patients were more likely to receive advanced respiratory support at some point during their admission (92.3% delay vs. 76.4% no-delay, *P *<0.01, Table 5). The delay group then required this advanced respiratory support for significantly longer than those in the no-delay group (median 4 vs. 3 days respectively, *P *= 0.04). There were no significant differences in the requirements for basic respiratory and cardiovascular support, advanced cardiovascular support, or renal support between the groups (Table 5).

**Table 5:** Organ support data

	Delay*N = 52*	No-Delay*N = 546*	*P*-value
**Advanced respiratory support days**			
*N receiving (%)*	*48 (92.3)*	*417 (76.4)*	*<0.01*
median (IQR)	4 (2 to 9)	3 (2 to 7)	0.04

**Basic respiratory support days**			
*N receiving (%)*	*31 (59.6)*	*341 (62.4)*	*0.67*
median (IQR)	2 (1 to 3.3)	2 (1 to 4)	0.98

**Advanced CV support days**			
*N receiving (%)*	*23 (44.2)*	*252 (46.3)*	0.89
median (IQR)	4 (2 to 8)	3 (2 to 6)	0.28

**Basic CV support days**			
*N receiving (%)*	*47 (90.4)*	*472 (86.4)*	0.42
median (IQR)	4 (2 to 8)	4 (2 to 7)	0.90

**Renal support days**			
*N receiving (%)*	*7 (13.4)*	*99 (18.1)*	*0.39*
median (IQR)	2 (2 to 6)	4 (2 to 12)	0.42

Only patients with critical care data set (n = 598) are included in analysis. Those not fulfilling the criteria for an organ support category (Table 1) were excluded from the analysis of duration of support for that system.

## Discussion

In this study we did not find an increased length of ICU stay, ICU mortality or hospital mortality rate in those patients whose admission to intensive care was delayed more than three hours. However, patients whose ICU admission was delayed had both a greater requirement for, and spent more time receiving, advanced respiratory support, that is, invasive mechanical ventilation.

The idea of a "golden hour" after major trauma, during which interventions made promptly after the initial injury carry the highest chance of preventing death, has been in use since the 1970's [17]. Although some controversy remains about its evidence base the concept has become well established [18]. Recent studies in critically ill patients who have sepsis have demonstrated that the early recognition of pathology and implementation of therapies, such as fluid resuscitation and appropriate antibiotics, can reduce mortality [19,20]. Such accelerated treatments now represent the accepted gold standard for a variety of conditions, such as myocardial infarction and stroke [21,22]. These studies suggest that time to treatment has a profound effect on outcome.

Whilst ICU admission can be seen as a surrogate for time to treatment in the critically ill, the fundamental factors determining outcome are likely to include specific interventions and the timely instigation of organ support. These do not always mandate immediate ICU admission and can be instigated on alternate sites, such as the ED or operating theatre suite, whilst an ICU bed is made available. This concept of "critical care without walls" is increasingly becoming accepted [23] and is in place at Charing Cross Hospital where the theatre suite is used to "board" patients pending ICU admission. Here patients are managed with ICU specialist input and it is feasible for all required treatments and interventions to be instigated. This practice may go some way to explaining why, in our study, delayed ICU admission did not prolong ICU admission or adversely effect mortality. Indeed, it is this practice of boarding that explains the large percentage of patients in the delay group admitted from the theatre suite as the patients are generally transferred and managed here whilst an ICU bed is created. This accounts for both the high percentage of delay admissions from theatres and their paradoxically low rates of surgery. Interestingly, the patients managed within the theatre suite had a trend towards a lower mortality compared to those patients managed in the ED/wards during the delay period. This suggests that the practice of boarding a patient in the theatre suite may be a better strategy than keeping the patients *in situ *on the ward if an ICU bed is not immediately available.

The incidence of delayed admission to the ICU in our study was 9.3%. Although there were no significant differences in demographics between the two groups, there was a trend toward a younger age within the delay group that may have had an effect on the outcome data. There were also baseline differences between the groups, with the delay group having significantly more patients with respiratory failure and fewer patients undergoing surgery immediately prior to admission. However, adjusting for these differences in linear and logistic regression analysis models did not alter the results.

Previous studies have described an association between prolonged hospital stay prior to ICU admission and increased mortality rates [24]. Other studies have described a high mortality amongst those ICU survivors discharged to the general ward at night [25], but few studies have examined the effect of a delay in ICU admission upon patient outcome. A North American study [26] examined 50,322 ICU transfers from the emergency department and defined a delayed admission as taking longer than six hours. Amongst the delayed group there was a significantly prolonged length of hospital stay, as well as increased ICU and hospital mortality. Subsequently, a European study [27] compared patients with community-acquired pneumonia admitted directly from the emergency department (no-delay) to the ICU, with patients admitted first to the general ward (delay). After adjusting for propensity score, an increased 28-day and hospital mortality were found in the delay group. Recently, a Brazilian study examined 401 ICU admissions and reported delay rates of 68.8% with a median duration of 17.8 hours [28]. These were associated with an increased length of ICU stay and mortality, with each hour of delay associated with a 1.5% increase in risk of ICU death. The high delay rate that they report is explained by their strict inclusion criteria (any patient not admitted immediately); however, the average delay was markedly longer in the Brazilian study (17.8 hours) compared to the 6 hours in our study. This may explain why, even after adjusting for baseline characteristics in a multivariate analysis, we found no association between length of delay and length of ICU stay or mortality.

Differences between our results and those of previous studies may also reflect differences in the management of these patients whilst an ICU bed is awaited. Within our institution two anaesthetic trainees manage these patients with senior input from the ICU specialist. Care directed by, or involving mandatory consultation from, an ICU specialist has been shown to improve outcomes [29], but may not be employed across all institutions and countries. A North American study reporting increased mortality associated with delayed in-patient transfer to an "open" ICU, in which all physicians had admitting rights, noted that delayed patients were less likely to have received a prompt physician review without specifying whether this was by an ICU specialist [30]. Another study reported that patients refused ICU admission due to lack of bed availability had a higher mortality if they were subsequently admitted to ICU but did not report how they were managed in the interim [31].

We found that delayed patients had a greater requirement for and duration of advanced respiratory support. This is likely to be due to the high rates of respiratory failure as the reason for ICU admission. Patients who require mechanical ventilation may be over-represented in the delay group as there is no facility to provide ventilation on the general ward, hence they would be transferred to ICU or the theatre suite immediately. This is not the case with other organ support strategies (for example, inotropes), which may sometimes be delivered on the general ward if an ICU bed is not available. This has important implications for clinical practice given that the delay group patients had a worse PaO_2_:FiO_2 _ratio during the first 24 hours after ICU referral compared to the no-delay group. Often these patients will receive mechanical ventilatory support from a portable transfer ventilator and/or an anaesthetic machine during the delay period and this may be detrimental to their respiratory physiology. It can be more difficult to provide lung protective ventilation (limiting pressure and volume, and providing positive end expiratory pressure) using these simple ventilators compared to a modern ICU ventilator. It has now become common practice within our hospital to manage patients with severe respiratory pathology waiting in the operating theatre suite by using an ICU ventilator whenever possible.

Limitations of our study must be considered. First, it was a retrospective analysis and organ support data were only collected for the final 21 months of the five-year period. However, ICU outcome data were available for all patients and hospital outcome data were only missing for <1% of patients. Although 1,609 patients were included over a five-year period, only 149 patients had a delayed admission and thus the power of the study is limited. In particular, it should be noted that the upper 95% confidence interval for ICU mortality in the delayed admission group is 2.0 and, therefore, we cannot exclude a potential doubling of mortality rates based on these data alone. It is possible that a larger patient cohort, with a larger number of delayed patients might detect significant differences in outcomes between delayed and non-delayed admissions. Using the data from this study, a power calculation suggests that approximately 25,000 patients would need to be studied to have 80% power to demonstrate a statistically significant difference in mortality rates. Clearly this would require a national multi-centre study and although it would have more power, any difference in outcome might also be affected by different management policies while a patient waited for ICU admission.

## Conclusions

In this study, managing critically ill patients "in-house" in a monitored environment when an ICU bed is unavailable is a viable option and did not prolong ICU stay or result in increased mortality. However, delayed ICU admission was associated with increased rates and a longer duration of mechanical ventilation and, therefore, robust strategies to provide lung protective ventilation need to be in place.

## Key messages

• Delayed ICU admission due to bed unavailability is common.

• Managing patients in the theatre suite whilst an ICU bed is arranged is a viable option.

• Patients whose ICU admission is delayed have increased rates and a longer duration of invasive mechanical ventilation.

## Abbreviations

APACHE II: Acute Physiology and Chronic Health Evaluation II; BiPAP: bi-level positive airway pressure; CVP: central venous pressure; CI: confidence interval; CPAP: continuous positive airway pressure; ED: Emergency Department; ETT: endo-tracheal tube; FiO_2_: fraction of inspired oxygen; GCS: Glasgow Coma Scale; ICU: Intensive Care Unit; IQR: inter-quartile range; OR: odds ratio; PaO_2_: partial pressure of oxygen in the blood; UK: United Kingdom

## Competing interests

The authors declare that they have no competing interests.

## Authors' contributions

DOC was involved in the design of the study, data collection, statistical analysis and interpretation, as well as drafting of the manuscript. PJ, EVH and MG were involved with data collection and interpretation, as well as drafting of the manuscript. MT was involved with design of the study, data collection and interpretation, as well as drafting of the manuscript. JS was involved with statistical analysis and interpretation, as well as drafting of the manuscript. AG conceived and supervised the study, contributed to its design, the data collection, statistical analysis and interpretation, and to the drafting of the manuscript. All authors have read and approved the final manuscript.
